# Insula activity in resting-state differentiates bipolar from unipolar depression: a systematic review and meta-analysis

**DOI:** 10.1038/s41598-021-96319-2

**Published:** 2021-08-20

**Authors:** Martin Pastrnak, Eva Simkova, Tomas Novak

**Affiliations:** 1 National Institute of Mental Health, Clinic, 250 67 Klecany, Czech Republic; 2 3rd Faculty of Medicine, Charles University, 100 00 Prague, Czech Republic

**Keywords:** Neuroscience, Biomarkers, Medical research

## Abstract

Symptomatic overlap of depressive episodes in bipolar disorder (BD) and major depressive disorder (MDD) is a major diagnostic and therapeutic problem. Mania in medical history remains the only reliable distinguishing marker which is problematic given that episodes of depression compared to episodes of mania are more frequent and predominantly present at the beginning of BD. Resting-state functional magnetic resonance imaging (rs-fMRI) is a non-invasive, task-free, and well-tolerated method that may provide diagnostic markers acquired from spontaneous neural activity. Previous rs-fMRI studies focused on differentiating BD from MDD depression were inconsistent in their findings due to low sample power, heterogeneity of compared samples, and diversity of analytical methods. This meta-analysis investigated resting-state activity differences in BD and MDD depression using activation likelihood estimation. PubMed, Web of Science, Scopus and Google Scholar databases were searched for whole-brain rs-fMRI studies which compared MDD and BD currently depressed patients between Jan 2000 and August 2020. Ten studies were included, representing 234 BD and 296 MDD patients. The meta-analysis found increased activity in the left insula and adjacent area in MDD compared to BD. The finding suggests that the insula is involved in neural activity patterns during resting-state that can be potentially used as a biomarker differentiating both disorders.

## Introduction

Depressive episodes are characteristic for both, major depressive disorder (MDD) and bipolar disorder (BD)^1–4^. Because the diagnostic criteria for depressive episodes are the same in both disorders^5,6^, establishing the correct diagnosis is difficult yet important as each requires different treatment^1,7,8^.

Several clinical characteristics and biological markers have been shown to increase the probability of diagnosing BD rather than MDD^9–11^, but none of them is included in diagnostic criteria, nor generally accepted without further exploration. The presence of mania in medical history remains the only accepted diagnostic marker that differentiates the two disorders. In addition to often unavailable or inconclusive psychiatric history, the diagnostic process is even more difficult given that episodes of BD depression compared to episodes of mania are longer, more frequent, and predominantly present at the beginning of BD^12–14^. Misdiagnosis is common and presents a dire problem: up to 40–60% of bipolar patients are misdiagnosed as unipolar and only 20% receive the correct diagnosis within the first year which may considerably increase risks of inappropriate drug prescription, switching to mania, prolonged illness duration, risk of recurrence, suicide and overall poorer treatment responses^7,15–18^. Hence, biologically relevant diagnostic markers of BD and MDD depressions would significantly improve the diagnostic process, yet are still unavailable.

While the pathological processes in psychiatric disorders (e.g. MDD and BD) are mostly unknown and the diagnosis is not data-driven and remains a clinical decision, the field of neuroimaging may contribute considerably in identifying biomarkers^19^. If found, rapidly growing advances in technology and data analysis may facilitate the utility of such biomarkers on levels of specificity and sensitivity that is relevant even for clinical use.

Resting-state functional magnetic resonance imaging (rs-fMRI) is a neuroimaging modality that focuses on low-frequency spontaneous fluctuations of blood oxygen level-dependent (BOLD) signal^20,21^. As opposed to the paradigm or task-based fMRI, in rs-fMRI the participants are usually asked to lay still in the scanner, with eyes closed or fixated on a cross-hair, and do nothing in particular. Depending on the used method of analysis, rs-fMRI enables to examine patterns of neural activity and connectivity at rest^22^. Rs-fMRI has a high potential for clinical use as it is non-invasive, safe, without task demands on the participants, relatively short, and well-tolerated even by severely depressed individuals^23^.

Presuming depressive episodes manifest with different activity in neurocircuitry in BD and MDD, these should be reflected in rs-fMRI scans. Numerous studies attempted to use rs-fMRI and various approaches to data analysis to find resting-state differences between patients with bipolar and unipolar depression. Mostly dorsolateral prefrontal, limbic, and midline structures were reported as differentiating the disorders in various measures of resting-state^3,24^, but no conclusive convergence of the results was reported yet. This inconsistency may arise from the diversity of methods used in the analyses, and the heterogeneity of the compared samples, which often differ across studies in size, clinical state, and comorbidities. Also, in fMRI studies, the cluster-wise inference (CWI) approach is often used in statistical analyses. This may present a problem as it was recently suggested that CWI in parametric statistical methods may lead to inflated false-positive rates, especially when lenient or uncorrected cluster-defining threshold is used^25^. Thus, heterogeneity may well result from false positives findings.

A quantitative meta-analytical approach may compensate for several of the aforementioned issues. It can attenuate the false-positive rates and the problem of sample comparability in terms of size and psychopathological variability^26,27^. To further enhance comparability across the studies, current psychosis should also be an exclusion criterion being more prevalent in BD compared to MDD^28^. In addition, psychotic mood episodes may be associated with additional brain abnormalities relative to nonpsychotic episodes and another confounding variable would have to be addressed. Finally, considering relatively indistinct diagnostic boundaries between bipolar disorder II and MDD, and metabolism^29^ and genetic^30,31^ studies suggesting a neurobiological difference between BD I and II, rather the narrow phenotype of BD I should be preferably investigated.

The uniformity of analytical methods is another challenging problem. Namely, there are only a few studies that compared depressive episodes in MDD and BD and used identical analytical methods. One way to overcome this is to focus on spatial information, e.g. brain-regions or their anatomical coordinates, of the reported differences in resting-state and include rs-fMRI studies regardless of the used analytical method^32–37^. However, this still limits the inclusion process to studies that do not restrict their rs-fMRI scans and following analysis to any particular brain regions. This approach also does not provide information on the nature of these differences such as functional connectivity or revealing functional networks. Instead, it provides identification of brain regions that have a high probability to contain information relevant for distinguishing BD from MDD depression.

The present meta-analysis aims to identify converging spatial information associated with alterations in spontaneous brain activity that differentiate patients with BD and MDD depression, using activation likelihood estimation (ALE)^36^. To enhance the validity of the results, the meta-analysis focuses on whole-brain rs-fMRI studies, with currently depressed, non-psychotic BD and MDD participants. If found, identified brain regions may be targeted for testing of new hypotheses and the development of neural biomarkers for the diagnosis of BD and MDD.

## Results

The inclusion process resulted in ten eligible studies^38–47^ with nine samples (two studies were performed on the same sample^40,41^) with a total of 234 BD and 296 MDD subjects. From the included studies, 7 experiments with 16 foci were extracted for the BD > MDD contrast and 6 experiments with 13 foci were extracted for the MDD > BD contrast.

Pooled analysis weighted by sample size of each study revealed only trend to younger age (BD: mean_pooled_ ± SD_pooled_ 30.4 ± 10.3 years; MDD: 32.5 ± 11.2 years; t = − 1.64, p = 0.10), and lower female proportion (BD: 54.1 ± 9.9%; MDD: 61.8 ± 8.8%; χ^2^ = 3.18, p = 0.07), but lower severity of depression (HAMD; BD: 23.8 ± 7.9; MDD: 25.5 ± 7.2; t = − 2.11, p = 0.03) in BD compare to MDD participants. Two studies included medication-free participants, while in two studies medication status was not specified. In remaining studies (n = 5; BD = 150, MDD = 194), comparable use of antidepressants (BD 38%, MDD 46%, p = 0.14), but higher rate of antipsychotics (BD 26%, MDD 4%, p ˂ 0.001), lithium (BD 12%, MDD 0%, p ˂ 0.001), and other mood stabilizers (BD 32%, MDD 2%, p ˂ 0.001) was found in BD patients.

All included studies had an upper-medium to high quality. The summary of the studies is detailed in Table 1.

**Table 1:** Studies included in the meta-analysis, methods of analysis, demographics, clinical data, medication, between-group contrasts, number of foci, main study outcome and study quality.

Study	Method of analysis	N	Age in years (SD)	Gender male/female	Depression severity (SD)	Medication					Contrast	Foci	Main outcome	Study quality
						AD	AP	Li	MS	Conf.				
Liu et al. (2012)^40^*	ALFF	21 BD	31 (8.46)	8/13	HAMD17 22.14 (3.18)	10	8	3	7	No	BD > MDD	1	R anterior insula	7
		21 MDD	33.3 (11.2)	9/12	HAMD17 22.52 (3.19)	15	3	0	1	No	MDD > BD	2	L posterior insula, L superior parietal lobule	
Liu et al. (2013)^41^*	ReHo	21 BD	31 (8.4)	8/13	HAMD17 22.14 (3.18)	10	8	3	7	No	BD > MDD	4	R dorsal anterior insula, R middle frontal gyrus, R posterior cerebellum, L anterior cerebellum	7
		21 MDD	33.3 (11)	9/12	HAMD17 22.52 (3.19)	15	3	0	1	No	MDD > BD	3	R posterior cingulate, R ventral anterior insula, R parahippocampal gyrus	
Liang et al. (2013)^39^	ReHo	17 BD	34.5 (9.7)	9/8	HAMD17 24.47 (4.9)	0	0	0	0	N/A	BD > MDD	1	Thalamus	8.5
		16 MDD	36 (9.4)	8/8	HAMD17 26.2 (4.98)	0	0	0	0	N/A	MDD > BD	N.S.	N.S.	
Li et al. (2017)^38^	DC	22 BD	28.7 (10.1)	9/13	HAMD17 20.8 (3.11)	11	10	N/A	10	Yes	BD > MDD	2	Bilateral precuneus, L cerebellum	9
		22 MDD	27.7 (8.7)	9/13	HAMD17 20.8 (2.97)	5	1	N/A	0	Yes	MDD > BD	1	L insula	
Yu et al. (2017)^42^	fALFF	13 BD	31.2 (19.5)	7/6	HAMD24 32.9 (7.3)	N/A	N/A	N/A	N/A	No	BD > MDD	N.S.	N.S.	5
		15 MDD	37.9 (7.1)	7/8	HAMD24 34,2 (3,8)	N/A	N/A	N/A	N/A	No	MDD > BD	4	L middle occipital gyrus, R middle temporal gyrus, L middle frontal gyrus, L medial frontal gyrus	
Zhang et al. (2017)^43^	fALFF	14 BD	33.8 (11)	6/8	HAMD17 18.54 (5.21)	0	0	0	0	n/A	BD > MDD	3	Bilateral putamen, L superior frontal gyrus	8
		13 MDD	33.5 (9.5)	6/7	HAMD17 20.15 (3.24)	0	0	0	0	n/A	MDD > BD	N.S.	N.S.	
Qiu et al. (2018)^44^	fALFF	28 BD	31.8 (12.8)	14/14	HAMD24 31 (7.92)	6	12	7	15	No	BD > MDD	3	L precuneus, L medial temporal gyrus, lingual gyrys	7
		47 MDD	38.1 (13.2)	20/27	HAMD24 30 (7.45)	25	2	0	2	No	MDD > BD	N.S.	N.S.	
Yao et al. (2018)^45^	ReHo	55 BD	27.2 (7.7)	22/33	HAMD17 20.18 (8.66)	20	10	N/A	17	No	BD > MDD	1	L frontal cluster	9
		76 MDD	26.5 (9.6)	19/57	HAMD17 22.43 (7.7)	32	1	N/A	0	No	MDD > BD	1	L temporal cluster	
Jiang et al. (2020)^46^	ReHo	24 BD	28,08 (9.55)	12/12	HAMD17 BD 22.04 (9.53)	9	0	0	1	Yes	BD > MDD	1	R superior/medial superior frontal gyrus	8
		28 MDD	30 (10.73)	10/18	HAMD17 MDD 26.21 (8.37)	11	0	0	0	Yes	MDD > BD	1	Bilateral precuneus/median cingulate/postcentral gyrus	
Liu et al. (2020)^47^	ReHo	40 BD	32.8 (7.44)	20/20	HAMD24 25.65 (4.9)	N/A	N/A	N/A	N/A	No	BD > MDD	N.S.	N.S.	7
		58 MDD	35.75 (9.9)	24/34	HAMD24 27.1 (4.6)	N/A	N/A	N/A	N/A	No	MDD > BD	1	R superior temporal gyrus	

*AD* antidepressant, *ALFF* amplitude of low frequency fluctuations, *AP* antipsychotics, *BD* bipolar disorder, *Conf.* whether medication was used as a confound, *DC* degree centrality, *fALFF* fractional ALFF, *HAMD* Hamilton Depression Rating Scale—17 items or 24 items, *Li* lithium, *MDD* major depressive disorder, *MS* mood stabilizer other than lithium, *N* number of subjects, *N/A* information not available, *N.S.* no significant group differences, *ReHo* regional homogeneity, *SD* standard deviation.

*Same sample.

**Figure 1:**
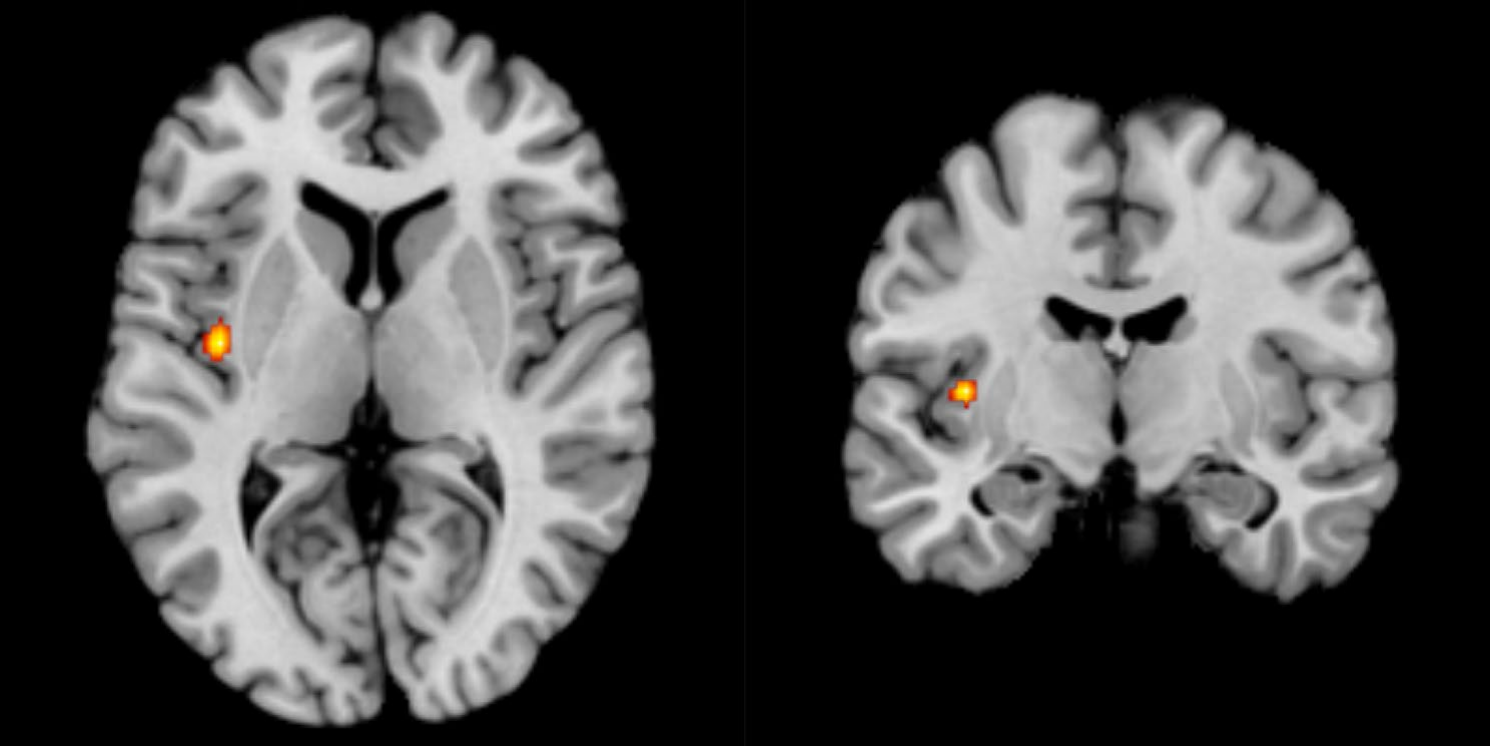
Axial and coronal view displaying the cluster of increased activity in unipolar depression compared to bipolar depression. Red to yellow: significant meta-analytic results (p < 0.05).

For the BD > MDD comparison, the ALE meta-analyses did not find any significant converging clusters (Table 2). For the MDD > BD comparison, the ALE meta-analysis identified a cluster covering the left insula and adjacent area overlapping the left claustrum (Table 2, Fig. 1).

**Table 2:** Meta-analysis results of the resting state activity differences in subjects with MDD compared to BD in both directions.

	Brain regions	Cluster volume (mm3)	Coordinates (MNI space)			Maximum ALE score
			X	Y	Z	
BD > MDD	N/A	N/A	N/A			0.0096
MDD > BD	Left insula, Left Claustrum	368	− 38.6	− 9.6	3.7	0.0129

Maximum ALE score represents the highest ALE value in the cluster.

*ALE* activation likelihood estimation, *BD* bipolar disorder, *MDD* major depressive disorder, *N/A* not applicable.

## Discussion

This ALE meta-analysis found consistently higher resting-state activity in left insula and adjacent area covering the claustrum in currently depressed MDD subjects relative to currently depressed BD subjects. No increased activity was found in BD subjects compared to MDD. The main implication of this finding is that the left insula may be involved in neural activity patterns during the resting state that can be potentially used as biomarkers differentiating both disorders.

In this study, only whole-brain rs-fMRI studies unrestricted to pre-selected brain-regions with currently depressed, nonpsychotic, and BD I and MDD participants were included. This led to a drop in included studies, but also increased the overall validity of the results. Assuming the main heterogeneity of findings in included studies resulted from the variability in imaging acquisition method and statistical analyses, ALE focused on spatial convergence was found to be the best meta-analytical option for this type of study^25,34^.

Cluster level FWE correction was applied in the meta-analysis which is regarded as the most appropriate method for statistical inference when using ALE^34^. In individual comparative studies, CWI is considered to be prone to false positive findings^25^. However, to use a voxel-wise inference in ALE would be too strict, especially when a rather small number of included studies was used. Therefore, to mitigate the CWI bias, in this ALE meta-analysis a recommended two-level thresholding with stricter thresholds was used^32^. First, on the voxel-level, the forming of clusters in the ALE image was thresholded on an uncorrected p < 0.0001. Second, the emergent clusters were tested on a threshold of p < 0.05 against 5000 permutations which were generated based on the original data sets. Thus, the surviving clusters should represent a true non-random convergence across the studies.

Next to the utility of the found regions as potential targets for future studies, the question arises what does the convergence of increased local activity in MDD relative to BD mean. ALE provides identification of non-random spatial convergence of included foci across the studies^35^. Higher activity in insula in MDD relative to BD as revealed by ALE may then represent an increased local neural activity, local over-connectivity, or increased connectivity with other regions of the brain. Furthermore, an increase in MDD relative to BD may not correspond to an increase relative to healthy subjects.

In support of our findings, there is emerging evidence of increased insular activity^38,40,48,49^ and connectivity^50–54^ in MDD relative to BD and controls. In addition, decreased regional metabolism in medication-free BD compared to controls was found in left dorsolateral and midline brain-regions, including the insula^55^. Moreover, while structural changes may not necessarily correspond with an increase or decrease in brain-region activity or connectivity, insular grey matter thickness was reported to be increased in MDD relative to controls^56,57^, and decreased in BD^58,59^ even before the onset of the illness. The decrease in grey matter volume in the insular and anterior cingulate cortex, both nodes of the salience network^60^, was also found in first-degree relatives of BD^61^. Reduction in gray matter volume in these structures, and potentially associated dysregulation of the salience network, may indicate an endophenotype of BD.

There are also studies that suggest opposite or mixed findings^41,62–65^. Nevertheless, they still indicate abnormalities located in the insula and structurally or functionally adjacent brain-regions and pathways (i.e. claustrum), which displayed differences in MDD relative to BD. Taken together, there is a growing body of evidence supporting that insular activity and connectivity is increased, or at least different, in resting-state MDD relative BD (and controls).

Increased activity and connectivity in insula in MDD may be interpreted in line with current knowledge about the functional properties of the structure and clinical observations. The insula is a multimodal integration region that evaluates the emotional and motivational salience of external and internal stimuli^66–68^. Typically, two functionally distinct regions are recognized in the insula^68^. First, visceral, somatosensory, vestibular, and motor inputs are relayed to the posterior insula. From there, they are forwarded to the second part, the anterior insula, where the re-representation of these inputs is integrated with emotional, cognitive, and motivational signals collected from cortical and subcortical regions. The anterior insula together with the anterior cingulate cortex and amygdala form the salience network (SN)^60,69,^ and is also interconnected to the dorsolateral prefrontal cortex and ventral striatum^66–68^. Furthermore, the anterior insula has been entitled the “limbic sensory area” connected with the anterior cingulate cortex, the “limbic motor area”^70–72^. In other words, the anterior insula is associated with visceral sensation, and the anterior cingulate cortex is associated with autonomic and emotional control. Lastly, the salience network has been implicated in detecting both interoceptive and external salient changes, signal for recruitment of additional processing, orient attention toward and react to salient stimuli, and switching between inward-oriented (e.g. default mode network (DMN)^73^) and externally directed cognition (e.g. central executive network(CEN)^60,69^.

Following this, increased activity in insula and adjacent neurocircuitry may correspond to clinical observations in which MDD patients report, among other MDD symptoms, increased introspective self-focus, rumination, and preoccupation on bodily states (somatic-muscular, respiratory, gastrointestinal, genitourinary, and autonomic symptoms)^53,74–76^. BD patients, on the other hand, display overall inhibition, emotional dampening, heaviness, tiredness of the body, slower thinking, inner tension, and fearfulness^74–76^. In other words, MDD patients are more focused on the inside and preoccupied with self-focus on their inner world (rumination) and bodily symptoms, and BD patients are more inhibited, blunted, and fearful.

From the perspective of functional connectivity and networks, the observed increased self-focus on inner states corresponds with the increased activity within DMN found in both MDD and BD^40,77–80^. However, increased activity within the SN^81^ and increased connectivity between the SN (specifically insula) and DMN was found in MDD^82–84^, which suggests an increased input from visceral and bodily states in MDD. In BD, the connectivity within and from the SN seems to be impaired, suggesting a dysregulated salience associated with a dysregulation of emotional control^85^. A recent study on the connectivity dynamics in neural networks also found that the switching rate of DMN was decreased in both MDD and BD relative to controls, suggesting an inability to navigate away from internal emotional and cognitive states^86^. However, MDD displayed a lower switching rate in SN and striatum relative to healthy controls and BD, which corresponds with increased insular activity on one hand, and decreased regulation of DMN by SN on the other^86^.

Increased activity in the claustrum in MDD was a rather surprising finding. No included study reported this region as differentiating the two disorders. One explanation is that claustrum was a component of larger significant clusters in the included studies, but more prominent structures nearby, such as insula or putamen, were labeled instead. Moreover, automated anatomical labelling atlases used in neuroimaging only rarely include claustrum (see AAL v3^87^). And conversely, it is possible that the true finding was insula and the claustrum was the result of an oversensitive atlas used in GingerALE software. Finally, the claustrum may be a result of a false-positive convergence of overlaps of surrounding significant structures. All these notions suggest caution in the interpretation of this finding.

The claustrum is still a poorly understood structure with high connectivity in the brain^88–91^. It is structurally and functionally connected with cortical and subcortical brain regions known to be compromised in neuropsychiatric disorders including the insula, anterior cingulate cortex, pre- and post-central gyrus, superior temporal gyrus, amygdala, and basal ganglia^90–93^; and likely acts as a relay node within several neural networks, which were proposed to be impaired in mental disorders^94^. Still, studies on claustrum in the context of neuropsychiatric disorders are sparse and its ontogenetic origin is still debated. From the sparse available research reduced volume^95^, hypoactivity^78^, and reduced metabolic activity^96^ in claustrum were reported in MDD; and reduced grey matter^97^ and increased metabolism^98^ was reported in BD. Currently, the claustrum is considered to belong to the insular cortex^99^, rather than the putamen and basal ganglia^100^. In this light, our findings suggest that distinct MDD and BD abnormalities in the claustrum are likely linked to altered insular functions and salience processing^101^.

The study has several limitations. The first cluster of limitations is related to the analyzed populations. MDD and BD differ in several characteristics that might affect the results but are difficult to control on both, the single-study level and the meta-analysis. Aggregated data showed different gender distribution and depression severity, and even age-comparable groups do not preclude dissimilar illness duration as the younger age at onset is more prevalent in BD. However, as MDD and BD groups were comparable in age, gender, and depression severity in all included studies, the difference on the whole-sample level likely had a limited impact on the ALE results.

Another issue that should be considered is the effect of different medications in MDD and BD. Two studies enrolled non-medicated participants, another two did not specify the medication status, but in the others, a higher rate of antipsychotics, lithium, and other mood stabilizers in BD groups was indicated. Especially antipsychotics are of relevance because they have been reported to lower or normalize insular activity in psychosis^65,102,103^. However, there are also studies showing opposite or no effect^104^. In addition, a former review has shown that in bipolar fMRI studies, both task-based and resting state, the antipsychotic medication had no altering effect on the results^105^. While the effect of the medication cannot be fully discarded without direct control, it is plausible to assume the lower insular activity in bipolar relative to unipolar depression is not a result of medication.

The next limitation is the small overall number of included experiments and the resulting sample sizes. This might have increased the likelihood that the ALE results were driven by a few experiments with a larger sample size^34^. Indeed, the largest study included by Yao et al.^45^ that represents one-fifth of the whole dataset (N = 131) is one of the two main contributors to the results; the second one is the study by Liu et al.^40^. And if either of the studies is removed from the dataset, the meta-analysis would yield no significant results. Importantly, other studies do contribute to the results as well, but the two studies are the leading contributors. Furthermore, Yao’s study is the only one with a mixed BD I and II sample and besides the sample size, this might be another issue to be considered.

The ALE method has limitations as well. One limitation is that ALE takes into account only reported foci, which may omit other significant regions within the reported clusters.

Another drawback of ALE is that studies with no reported significant findings are not accounted for in the calculation. There were studies in both contrast groups (BD > MDD and MDD > BD) that indeed did not report significant findings in both directions (see Table 1). This may have led to an overestimation of the findings.

The exclusion of ROI-to-Voxel or ROI-to-ROI studies, which was justified to accommodate ALE requirements, also presents a potential bias of non-reporting of significant results. On the other hand, the risk of bias by including such studies would render the results highly unreliable.

Finally, as stated above, ALE does only inform of non-random convergence of findings. This cannot fully dismiss the risk of identifying meaningless false-positive convergences (e.g. convergence in white matter, ventricles, etc.). In other words, it may pinpoint an insignificant region only because it was surrounded by true significant structures.

In conclusion, the current meta-analysis applied the ALE method to identify convergence of results of resting-state fMRI studies that compared MDD and BD currently depressed individuals. The results showed that the left insula and potentially claustrum might be involved in neural activity patterns during resting state that can be used as biomarkers differentiating both disorders. The finding is in line with clinical observations in which MDD patients display more pronounced symptoms (ruminative self-focus, focus on bodily symptoms) associated with abnormal functions of neurocircuitry involving insula and adjacent brain regions than BD patients. Future studies should confirm our findings on a larger sample, e.g. on more than ten datasets, and explore their sensitivity and specificity in differentiating both disorders.

## Methods

Prior to the study initiation, the study was pre-registered on PROSPERO (https://www.crd.york.ac.uk/prospero/) with project ID CRD4201811443. The study was conducted in compliance with the PRISMA (Preferred Reporting Items for Systematic Reviews and Meta-analyses) guidelines for systematic review and met-analysis^106^.

### Search strategy and selection of studies

A systematic search was conducted on the PubMed, Web of Science (WOS) and Scopus databases. A secondary search was conducted on the Google Scholar (GS) database. All searches were confined from January 2000 to August 2020 and performed by MP. In PubMed, WOS and Scopus titles, keywords, and abstract searches were conducted using the following terms: (bipolar OR bipolar disorder) AND (unipolar OR depression OR depressive episode OR major depressive disorder OR depressive disorder) AND (fMRI OR functional magnetic resonance) AND (rest OR resting state). In GS, a combination of the same keywords was used and the search was limited to the first 200 articles sorted by relevance. After removing duplicates, the systematic search in PubMed, WOS and Scopus identified 1508 studies. Two researchers (MP, ES) independently screened the titles, keywords, and abstracts of the studies for relevance and filtered out non-English, non-peer-reviewed, and unpublished articles. Sixty-two articles passed the initial screening of PubMed, WOS and Scopus studies and their reference lists were searched for additional potential studies with no new results. Screening of GS studies did not identify any additional articles. Two researchers (MP, ES) evaluated the full-texts of the 62 articles on the following criteria for study inclusion: (1) age of participants 18+, (2) reported Montreal Neurological Institute (MNI) or Talairach whole-brain contrasts comparing BD and MDD subjects in rs-fMRI, (3) both compared samples currently depressed, (4) moderate severity of depression when enrolled (MADRS ≥ 20, HAMD ≥ 17), (5) at least ten subjects per group. Subsequently, excluding criteria were applied: (1) reported psychiatric or neurological comorbidity, (2) study included only bipolar type II participants, (3) seasonal depression, dysthymia, (4) psychosis, (5) only task-based MRI experiments reported, (6) independent component analysis (ICA) performed and only a specific component was examined, (7) only a priori region of interest (ROI) analysis or seed-based functional connectivity analysis performed. Potential disagreements between the evaluators were to be resolved by the third author (TN).

Exclusion criteria 6 and 7 were based on the recommendation from BrainMap (see http://brainmap.org/taxonomy/criteria.html) according to which studies that intentionally restrict the image acquisition and/or analysis to pre-selected ROI (or a specific ICA component) that is smaller than the whole brain should be excluded. Because the ALE algorithm assumes that activity in each voxel in the brain is equally likely to occur, foci from pre-selected ROIs may be reported as significant and result in an increase in false-positive rate. Studies, which were not based on BOLD (e.g. perfusion studies), were excluded as well.

**Figure 2:**
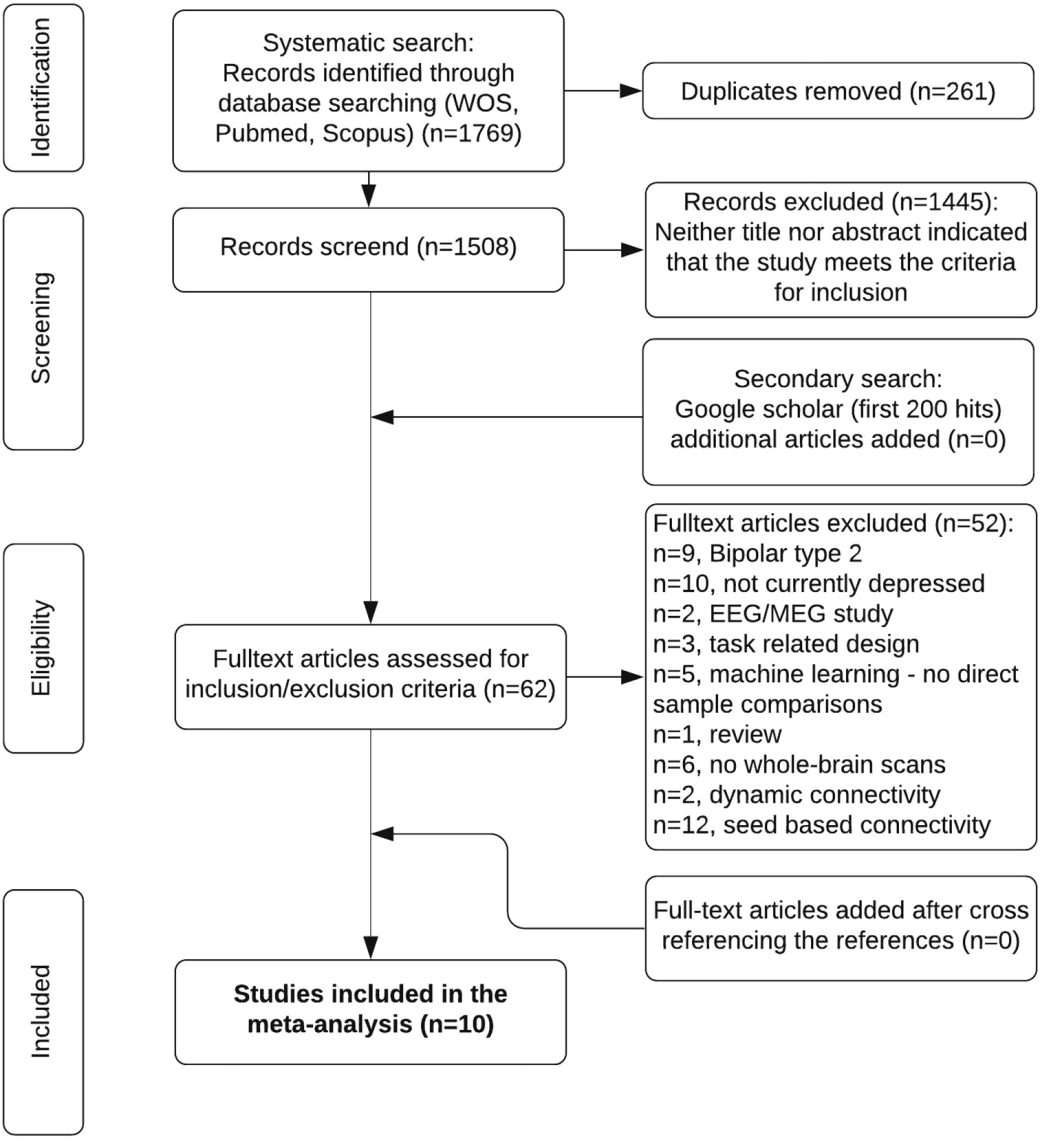
PRISMA flowchart showing the process of identifying the articles included in the meta-analyses.

From the 62 studies, 52 were excluded after full text read. The remaining ten rs-fMRI studies used amplitude of low frequency fluctuation (ALFF)^40^ and fractional ALFF (fALFF)^42–44^, regional homogeneity (ReHo)^39,41,45–47^, and degree centrality (DC)^38^. The process is summarized in the PRISMA flowchart shown in Fig. 2.

### Study quality assessment

Individual study quality was assessed with a 10-point checklist (Supplementary material S1) based on previous meta-analytic studies^107–109^ for the quality of the sample sizes, diagnostic procedures, demographic and clinical parameters, the fMRI acquisition parameters, analysis method, and the quality of reported results. The quality of every included paper was reviewed by two authors independently (MP, ES). Ratings were compared and inconsistencies were discussed in order to obtain a consensus score.

### Data extraction

From each included article MNI coordinates (e.g. foci) of reported peak values of significant between-group differences corrected for multiple comparisons were extracted (MP). No study used Talairach coordinates. Coordinates were assigned to two subgroups based on directionality to avoid that opposite findings across studies enhanced each other in the following analysis. The first group contained findings of increased DC, ReHo, ALFF, and fALFF in BD compared to MDD (BD > MDD). In the same manner, the second group included findings of increased resting-state measures in MDD compared to BD (MDD > BD). In each directionality subgroup, the lower n of the two samples (BD or MDD) from each study was assigned in the foci datasets, which were used in the meta-analytic calculation. The actual n of MDD and BD samples was used in calculations of heterogeneity (pooled analyses) of demographic and clinical variables across the studies. As each study may report findings in one or both directionalities and use different methods of analysis, findings in one directionality and/or analysis were referred to, according to the convention, as experiments. Hence, any study may contain coordinates from one or more experiments. Foci from different experiments conducted on the same samples, either within one study or in different studies, were grouped together. This was the case of two studies^40,41^ which compared the same MDD and BD sample, but used different analytical methods (e.g. ALFF and ReHo). Their findings for each directionality were grouped together in the main ALE meta analysis calculations and the samples were handled as one study in pooled analyses.

Scanning parameters of included rs-fMRI studies are available in Supplementary material S2.

### Statistical analysis

GingerALE v3.02 (www.brainmap.org) software was used for the meta-analysis. After entry, the coordinates were masked using the conservative standard mask from GingerALE. ALE method treats each foci in a given experiment as a three-dimensional Gaussian probability distribution that represents the spatial uncertainty associated with the coordinate. The Full Width at Half Maximum (FWHM) of these Gaussian functions was determined automatically by GingerALE based on the number of subjects per experiment^34^. This accommodated the assumption that a larger sample size in an experiment provides more reliable approximations of the true activation effect and was therefore modeled by smaller Gaussian distributions and vice versa. Next, a model activation (MA) map was generated for each experiment by combining the probability distributions. To limit the cumulative effect of multiple foci close to each other within a given experiment, the non-additive approach was applied, which generated MA maps by taking the maximum probability across overlapping Gaussians^37^. Final ALE scores were computed on a voxel-by-voxel basis by taking the union across these MA maps. The resulting ALE image was thresholded on a cluster forming threshold of p < 0.0001 and corrected with a family-wise error correction with cluster-level inference threshold at p < 0.05 and 5000 permutations^32^.

The main ALE meta-analysis was conducted for both directionality subgroups (e.g. BD > MDD and MDD > BD). Supplementary ALE meta-analytic calculations were also conducted for each modality and directionality subgroup. Datasets with foci and ALE calculations are available at https://osf.io/e7y6c/ (Open Science Framework).

All analyses were calculated in MNI space. Anatomical labels were automatically assigned by GingerALE. Visualizations were created using Mango version 3.0.4 (http://ric.uthscsa.edu/mango/) and a high-resolution anatomical template with isotropic voxels in MNI space as distributed with GingerALE.

## Supplementary Information

Supplementary Information 1.

Supplementary Information 2.

Supplementary Information 3.

## Data Availability

The data that support the findings of this study are freely available at https://osf.io/e7y6c/ (Open Science Framework) or by request from the corresponding author, MP.
