# Supplementary material for: Insula activity in resting-state differentiates bipolar from unipolar depression: a systematic review and meta-analysis
Source: Sci Rep. 2021 Aug 20;11:16930. doi: 10.1038/s41598-021-96319-2 (PMC8379217; doi:10.1038/s41598-021-96319-2)
Supplement: Supplementary file 1 — Supplementary Information 1. [file 41598_2021_96319_MOESM1_ESM.docx]

**Insula activity in resting state differentiates bipolar from unipolar depression - a systematic review and meta-analysis**

Authors: Martin Pastrnak, Eva Simkova, Tomas Novak

Supplementary material S1

**Quality assessment checklist**

**Category 1: Subjects**

1. Patients evaluated prospectively; specific diagnostic criteria applied; demographic data reported.

2. Comparison subjects evaluated prospectively; psychiatric and medical illnesses excluded; demographic data reported.

3. Important confounds (e.g. age, gender, trauma type, illness duration, medication status, comorbidity, illness severity) controlled either by stratification or statistically.

4. Sample size per group > 10.

**Category 2: Methods for image acquisition and analysis**

5. Whole brain analysis automated with no apriori regional selection.

6. Coordinates reported in a standard space.

7. Imaging technique clearly enough described to be reproduced.

8. Measurements clearly enough described to be reproduced.

**Category 3: Results and Conclusions**

9. Statistical parameters provided for significant and important non-significant differences.

10. Conclusions consistent with results; limitations discussed.

Score (0 / 0.5 / 1):
