# Supplementary material for: Insula activity in resting-state differentiates bipolar from unipolar depression: a systematic review and meta-analysis
Source: Sci Rep. 2021 Aug 20;11:16930. doi: 10.1038/s41598-021-96319-2 (PMC8379217; doi:10.1038/s41598-021-96319-2)
Supplement: Supplementary file 2 — Supplementary Information 2. [file 41598_2021_96319_MOESM2_ESM.docx]

**Insula activity in resting state differentiates bipolar from unipolar depression - a systematic review and meta-analysis**

Authors: Martin Pastrnak, Eva Simkova, Tomas Novak

Supplementary material S2

**Parameters of resting state functional magnetic resonance imaging scans in individual studies**

Liu et al. 2012

magnetic resonance field strength: 3T

TR=2000ms

TE=30ms

scan duration: 8min

FWHM: not reported/performed (this step is still disputed in ReHo and ALFF/fALFF preprocessing)

spatial normalization: MNI 3x3x3mm

multiple comparison correction method: p<0.01 AlphaSim corrected

Liang et al., 2012

magnetic resonance field strength: 1.5T

TR=3000ms

TE=60ms

scand duration: 6min 24s

spatial normalization: MNI 3x3x3mm

FWHM: 8x8x8mm

multiple comparison correction method: Monte Carlo (AlphaSim)

Liu et al., 2013

magnetic resonance field strength: 3T

TR=2000ms

TE=30ms

scan duration:8min

spatial normalization: MNI

FWHM: 4x4x4 mm (smoothing of ReHo maps)

multiple comparison correction method: Monte Carlo

Li et al., 2017

magnetic resonance field strength: 3T

TR=2000ms

TE=3.711ms

scan duration: 8min

spatial normalization: MNI

FWHM: 6x6x6 mm (smoothing of the DC maps)

multiple comparison correction method: FWE

Yu et al., 2017

magnetic resonance field strength: 3T

TR=2000ms

TE=30ms

scan duration:6min

spatial normalization: MNI 3x3x3 mm

FWHM: 6x6x6 mm

multiple comparison correction method: Monte Carlo

Zhang et al., 2017

magnetic resonance field strength: 3 T

TR=2000ms

TE=30ms

scan duration:7min 4s

spatial normalization: MNI 3x3x3 mm

FWHM: 6x6x6 mm

multiple comparison correction method: p<0.05 Gaussian Random Field theory correction

Qiu et al., 2018

magnetic resonance field strength: 3T

TR=2000ms

TE=30ms

scan duration:6min 46s

spatial normalization: MNI 3x3x3 mm

FWHM: 8x8x8 mm

multiple comparison correction method: voxel-wise *P* < 0.001 (uncorrected)

Yao et al., 2018

magnetic resonance field strength: 3T

TR=2000ms

TE=40ms

scan duration:6min 40s

spatial normalization: 3x3x3mm MNI

FWHM: 6x6x6 mm

multiple comparison correction method: p<0.05 Bonferoni correction

Jiang et al., 2020

magnetic resonance field strength: 3T

TR:2000ms

TE:30ms

scan duration: 6min 40s

spatial normalization: MNI

FWHM: 6x6x6 mm

multiple comparison correction method: p<0.05 Bonferoni correction

Liu et al., 2020

magnetic resonance field strength: 3T

TR:2000ms

TE:30ms

scan duration: 7min 4s

spatial normalization: 3x3x3 MNI

FWHM: 6x6x6 mm

multiple comparison correction method: p<0.01 AlphaSim correction
